# Cavity-less on-chip optomechanics using excitonic transitions in semiconductor heterostructures

**DOI:** 10.1038/ncomms9478

**Published:** 2015-10-19

**Authors:** Hajime Okamoto, Takayuki Watanabe, Ryuichi Ohta, Koji Onomitsu, Hideki Gotoh, Tetsuomi Sogawa, Hiroshi Yamaguchi

**Affiliations:** 1 NTT Basic Research Laboratories, Nippon Telegraph and Telephone Corporation, Atsugi 243-0198, Japan; 2 Department of Physics, Tohoku University, Sendai, Miyagi 980-8578, Japan

## Abstract

The hybridization of semiconductor optoelectronic devices and nanomechanical resonators provides a new class of optomechanical systems in which mechanical motion can be coupled to light without any optical cavities. Such cavity-less optomechanical systems interconnect photons, phonons and electrons (holes) in a highly integrable platform, opening up the development of functional integrated nanomechanical devices. Here we report on a semiconductor modulation-doped heterostructure–cantilever hybrid system, which realizes efficient cavity-less optomechanical transduction through excitons. The opto-piezoelectric backaction from the bound electron–hole pairs enables us to probe excitonic transition simply with a sub-nanowatt power of light, realizing high-sensitivity optomechanical spectroscopy. Detuning the photon energy from the exciton resonance results in self-feedback cooling and amplification of the thermomechanical motion. This cavity-less on-chip coupling enables highly tunable and addressable control of nanomechanical resonators, allowing high-speed programmable manipulation of nanomechanical devices and sensor arrays.

## Abstract (web summary)

Integrated optomechanical systems can be created by combining semiconductor optoelectronic devices and nanoscale resonators. Here, the authors demonstrate a semiconductor modulation-doped heterostructure-cantilever that achieves efficient optomechanical transduction without the need for an optical cavity.

## Graphical abstract

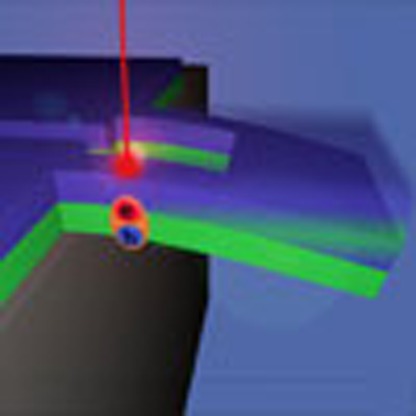

Optical control of nanomechanical resonators has been widely demonstrated in cavity-integrated optomechanical systems^1^,^2^,^3^,^4^,^5^,^6^,^7^,^8^,^9^,^10^,^11^,^12^. In these schemes, photons confined in a cavity cause radiation pressure or photothermal backaction onto a mechanical resonator, which acts in a time delay with respect to the mechanical motion. This leads to self-feedback that can cool and amplify the mechanical motion when the photon energy is red or blue detuned from the cavity resonance^1^,^2^,^3^. Although the cavity optomechanics allows highly tunable control of a single nanomechanical resonator, it cannot be straightforwardly extended to integrated nanomechanical systems, such as nanomechanical circuits and sensor arrays^13^,^14^,^15^,^16^,^17^,^18^,^19^. This is because it needs delicate cavity operation, including tapered fibre access, coupling adjustment, cavity stabilization and fine detuning^1^,^2^,^3^. Therefore, alternative approaches are highly demanded in order to practically apply the optical control capability to integrated nanomechanical systems. One approach being developed is based on optomechanical crystals in which photons and phonons are co-localized using band gap^20^. The two-dimensional crystals have been recently realized^21^, where the optomechanical arrays would eventually be connected by on-chip waveguides. However, such systems require strong link between structural dimensions and the optical wavelength to spatially collaborate the optical mode with the mechanical mode. Thus, a more scalable ‘cavity-less' scheme that also allows free-space optical access is strongly desired to be developed.

Cavity-less optomechanical coupling based on the free-space optical access has recently been demonstrated in several III–V semiconductor micro/nanomechanical systems^22^,^23^,^24^. Yeo *et al*. and Montinaro *et al*. have reported the coupling of exciton and mechanical motion in quantum dot-nanowire hybrid systems^22^,^23^. They were inspired by the theoretical proposal, by Wilson-Rae *et al*., of sideband cooling using a quantum two-level systems^25^. However, the cooling has not been experimentally implemented because the linewidth of the exciton resonance is orders of magnitude larger than the mechanical resonance frequency, which makes it difficult to achieve efficient optomechanical self-feedback through the phonon-exciton parametric coupling. As a different approach, Okamoto *et al*. have reported the opto-piezoelectric backaction through electron–hole (*e*–*h*) pairs in an *n*/*i*-GaAs bilayered cantilever^24^. Spatial separation of electrons and holes due to the built-in electric field causes retarded opto-piezoelectric backaction, which leads to self-feedback on the mechanical resonator. However, the broad absorption edge screens the sharp exciton resonance. This results in inefficient optomechanical self-feedback that prevents the realization of mode cooling.

Here we demonstrate exciton-mediated strong opto-piezoelectric backaction, which allows cavity-less cooling and amplification of a mechanical mode based on the free-space optical access. We use a cantilever with a GaAs/AlGaAs modulation-doped heterostructure, in which photoexcited *e*–*h* pairs are bound to form excitons by Coulombic interactions^26^. This structure results in sharply defined exciton resonance (FWHM≡Δ*E*_e_≃1 meV and the equivalent quality factor *Q*_e_≡*E*_e_/Δ*E*_e_=1,500), which plays the similar role as an optical cavity. The photon energy detuning from exciton resonance activates the optomechanical self-feedback as in the case of standard cavity optomechanics. It should be noted that the backaction force is not caused by the phonon-exciton parametric coupling^25^ but by the carrier-induced piezoelectric force^24^ in our sample. Since Δ*E*_e_ is five orders of magnitude larger than the mechanical resonance frequency (*f*_0_=*ω*_0_/2*π*=386.68 kHz), the parametric coupling is negligibly small. Nevertheless, efficient optomechanical self-feedback is induced via the opto-piezoelectric effect through excitons. This is because the corresponding time delay (*τ*=0.4–0.8 μs), which comes from the spatial confinement of free *e–h* pairs to form their bound state, is comparable to the mechanical oscillation period. This retarded backaction realizes cooling as well as amplification of the mechanical mode without any optical cavities. Cooling of the fundamental mechanical mode (*Q*_m_=5,600) by a factor of 2 from the bath temperature (*T*=9.2 K) is achieved with laser irradiation of 3 μW.

## Results

### Opto-piezoelectric driving

**Figure 1:**
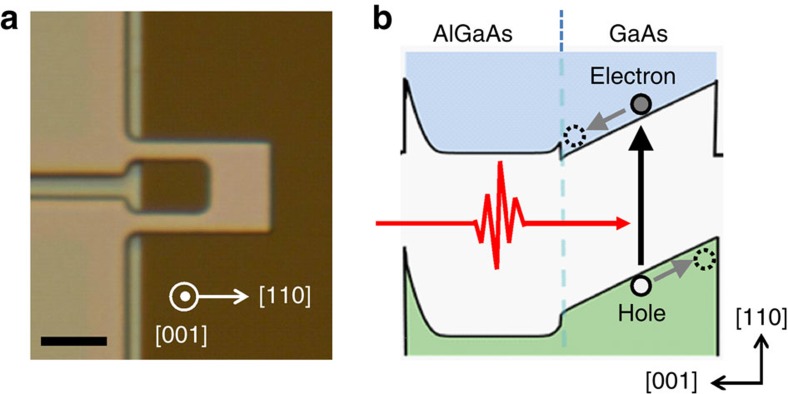
A semiconductor modulation-doped heterostructure–cantilever hybrid system. (**a**) Optical micrograph of the U-shaped cantilever with a GaAs/AlGaAs modulation-doped heterostructure (scale bar, 10 μm). The fundamental mechanical mode of the [110]-oriented cantilever corresponds to the out-of-plane vibration along [001]. This crystal orientation enables us to utilize opto-piezoelectric backaction in the GaAs-based mechanical system^24^,^26^. (**b**) Calculated energy-band diagram, in which the separation of photoexcited electrons and holes is schematically drawn. The band-gap energy is about 1.516 eV at cryogenic temperatures.

**Figure 2:**
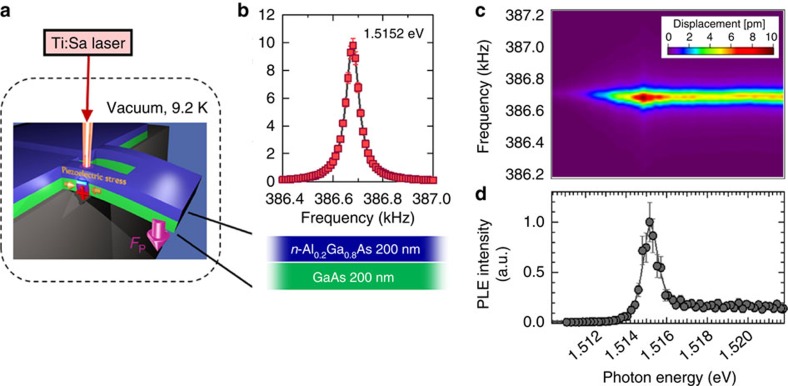
Optomechanical driving with modulated illumination. (**a**) Schematic drawing of the opto-piezoelectric effect mediated by *e–h* pairs. Separation of electrons and holes due to the built-in electric field leads to the dipole moment (indicated by a pair of minus and plus), which generates piezoelectric compressive stress (indicated by the yellow arrows). This bends the cantilever as backaction force *F*_p_ acts on it (indicated by the purple arrow). (**b**) Frequency response of the cantilever when the photon energy was tuned to the exciton resonance (*E*_e_=1.5152, eV) with the laser power fixed to 0.5 nW. (**c**) Photon-energy dependence of the frequency response under modulated illumination. (**d**) Photoluminescence excitation (PLE) spectrum, which displays a sharp exciton resonance at 1.5152, eV. The error bars are due to the systematic uncertainties in the PLE intensity. The equivalent quality factor of the exciton resonance is *Q*_e_≡ *E*_e_/Δ*E*_e_=1,500, where Δ*E*_e_ is the full width at half maximum of the exciton resonance. a.u., arbitrary unit.

Figure 1a shows an optical micrograph of the cantilever. The GaAs/AlGaAs modulation-doped heterostructure provides not only a built-in electric field as shown in Fig. 1b, but also sharp excitonic absorption at around 1.515 eV at cryogenic temperatures, which can be confirmed through the intensity *I* of the photoluminescence excitation (PLE) spectrum (Fig. 2d). These characteristics are necessary in order to obtain the enhanced opto-piezoelectric backaction from bound *e–h* pairs, namely excitons, via the time-delayed separation of electrons and holes. To generate *e–h* pairs, we focused a wavelength-tunable Ti:Sa laser on a leg of the U-shaped cantilever where optomechanical coupling is effectively induced via strain (Fig. 2a).

The generation of opto-piezoelectric backaction was confirmed by the following driven measurement: the intensity of the Ti:Sa laser was sinusoidally modulated, while the frequency was swept around the fundamental mechanical mode. The frequency response of the cantilever was measured in a vacuum (5 × 10^−5^ Pa) by Doppler interferometry with a He:Ne laser (633 nm). This laser was focused near the free-edge of the cantilever where larger displacement results. The frequency response when the photon energy was set to the exciton resonance energy (*E*_e_=1.5152, eV) shows the Lorentzian resonance (Fig. 2b), which displays the optically driven fundamental mechanical mode. The photon-energy dependence of the frequency response reveals that the vibration amplitude is maximized at the exciton resonance energy (Fig. 2c). This indicates the resonator is driven by the opto-piezoelectric process mediated by *e–h* pairs with the following mechanism^24^: photo-excited electrons and holes in the GaAs layer are separated by the built-in electric field along the thickness direction (Fig. 1b), generating a dipole moment (Fig. 2a). This leads to piezoelectric compressive stress, resulting in a bending moment as if the cantilever is subjected to vertical backaction force *F*_p_ (Fig. 2a). This backaction force drives the cantilever when it is periodically modulated with the mode frequency by intensity-modulated illumination. The vibration amplitude is sensitive to the photon energy (Fig. 2c) because *F*_p_ is proportional to the number of *e–h* pairs *N* that contribute the opto-piezoelectric backaction. Thanks to the *Q*_m_-enhanced resonant amplitude, this driven measurement enables a fine mechanical probe of the optical transitions.

### Cavity-less cooling and amplification

**Figure 3:**
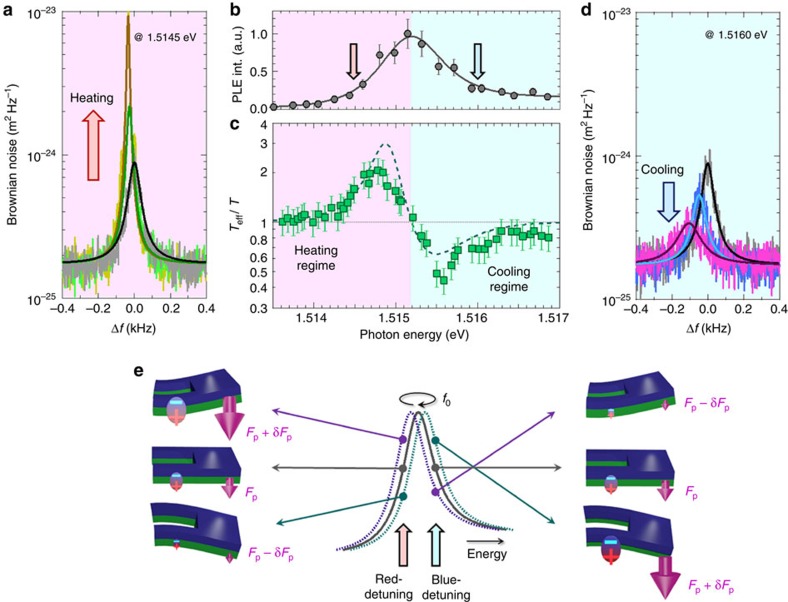
Amplification (heating) and cooling of the mechanical mode with constant illumination. (**a**) Brownian displacement noise power spectrum for *P*_ex_=0 (black), 1.82 (green) and 2.95 μW (yellow) at 1.5145, eV (red detuning). The solid line is the Lorentzian fit. The horizontal axis show the shift from *f*_0_. (**b**) PLE spectrum in the vicinity of the exciton resonance. The red and blue arrows indicate the detuning point for **a** and **d**, respectively. (**c**) The photon-energy dependence of normalized mode temperature *T*_eff_/*T* for *P*_ex_=1.19 μW. The error bars are based on the fitting of the Brownian noise power spectrum to the Lorentzian function. The broken line is a fit with the theoretical form *T*_eff_/*T*=(1+*CdI*/*dE*)^−1^, where *dI*/*dE* is the derivative of the PLE intensity *I*. (**d**) Brownian displacement noise power spectrum for *P*_ex_=0 (black), 1.19 (blue) and 5.16 μW (purple) at 1.5160, eV (blue detuning). (**e**) Schematic drawing of the strain-modulated exciton resonance and the strain-dependent opto-piezoelectric backaction for red and blue detuning. A pair of minus and plus indicates the dipole moment induced by the separation of electrons and holes. The purple arrow indicates the strain-dependent opto-piezoelectric backaction force.

The opto-piezoelectric backaction results in self-feedback on the thermomechanical vibration when the cantilever is constantly illuminated with the Ti:Sa laser with the slight energy detuning from the exciton resonance. Figure 3a,d show the change in the Brownian displacement noise power spectrum of the cantilever when the photon energy is red (1.5145, eV) and blue (1.5160, eV) detuned, respectively (see also the magnified PLE spectrum in Fig. 3b). For the red detuning, increasing laser power *P*_ex_ increases the vibration amplitude with narrowing the linewidth of the resonance, which corresponds to the damping factor (Fig. 3a). Increasing *P*_ex_ also increases the area of the noise power spectrum that reflects mode temperature *T*_eff_, indicating heating of the mechanical mode. In contrast, for the blue detuning, increasing *P*_ex_ decreases both the amplitude and area with linewidth widening, indicating cooling of the mechanical mode (Fig. 3d). The photon-energy dependence of normalized mode temperature *T*_eff_/*T* for *P*_ex_=1.19 μW clearly shows heating (amplification) in the red-detuning region and cooling in the blue-detuning region (Fig. 3c). This indicates the self-feedback is caused by the strain-induced change in the exciton resonance (Supplementary Note 5), where the feedback efficiency depends on the slope of the absorption (PLE) spectrum.

As shown in Fig. 3e, the exciton resonance is modulated by strain via the deformation potential^27^. Since the excitons sit in the lower half of the cantilever (in the GaAs layer) the exciton resonance is red shifted when the cantilever bends upwards (purple broken curve in Fig. 3e) and blue shifted when it bends downwards (blue broken curve in Fig. 3e). Therefore, when the photon energy is red detuned, the upward bending increases the dipole moment with an increase in absorption, leading to an increase in the backaction force, that is, *F*_p_+*δF*_p_ (left top schematic in Fig. 3e). Conversely, the downward bending decreases the dipole moment with a decrease in absorption, leading to a decrease in the backaction force, that is, *F*_p_−*δF*_p_ (left bottom schematic in Fig. 3e). That is, the backaction force is dependent on strain and the displacement, *z*. In this red-detuned case, the sign of force gradient ∇*F*_p_ (=∂*F*_p_/∂*z*) is negative. This negative ∇*F*_p_ and the corresponding time delay 
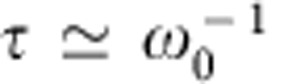
 (Supplementary Fig. 1 and Supplementary Note 4) result in the efficient amplification effect around the mechanical resonance frequency *ω*∼*ω*_0_ while reducing the damping factor (see equation (1) in the next paragraph). In contrast, when the photon energy is blue detuned, the upward bending decreases the backaction force, that is, *F*_p_−*δF*_p_ (right top schematic in Fig. 3e), whereas the downward bending increases it, that is, *F*_p_+*δF*_p_ (right bottom schematic in Fig. 3e). Therefore, ∇*F*_p_ is positive in this blue-detuned case and it leads to the efficient damping effect. Note that the above detuning dependence is the opposite of sideband amplification/damping^1^,^25^. In this excitonic optomechanics, the feedback is caused by strain-induced modulation of the number of *e–h* pairs. Thus, the polarity of the feedback is determined by the sign of the slope in the absorption (PLE) spectrum and also by the sign of the piezoelectric coefficient^27^.

The damping factor modified by this opto-piezoelectric backaction Γ_eff_ can be given by^24^


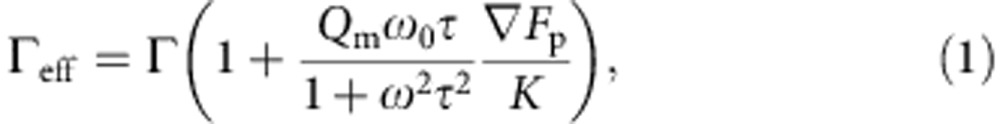


where Γ is the bare damping factor, *K* is the bare spring constant, and *τ* is the delay time for the opto-piezoelectric backaction (Supplementary Note 1). Modified mode temperature *T*_eff_ is inversely proportional to Γ_eff_ as^24^


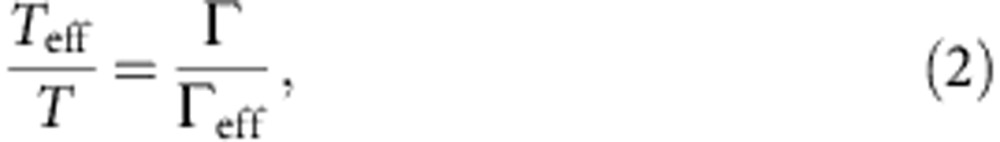


where *T* is the bath temperature. In the excitation regime of *P*_ex_<10 μW, *F*_p_ is proportional to *P*_ex_, which is confirmed by the vibration amplitude in the driven measurement (Supplementary Fig. 2). This means that the band flattening caused by the screening effect is negligibly small in this power regime; thus, ∇*F*_p_ in equation (1) is proportional to *P*_ex_. Since the backaction force depends on the strain-induced change in the number of *e–h* pairs for a fixed laser power, ∇*F*_p_ is also proportional to the slope of the PLE spectrum. Therefore, the mode temperature reflects d*I*/d*E*, following the theoretical form *T*_eff_/*T*=Γ/Γ_eff_=(1+*C*d*I*/d*E*)^−1^ (broken line in Fig. 3c; Supplementary Note 1), where *E* is the detuning energy from the excition resonance and *C* is a coefficient.

**Figure 4:**
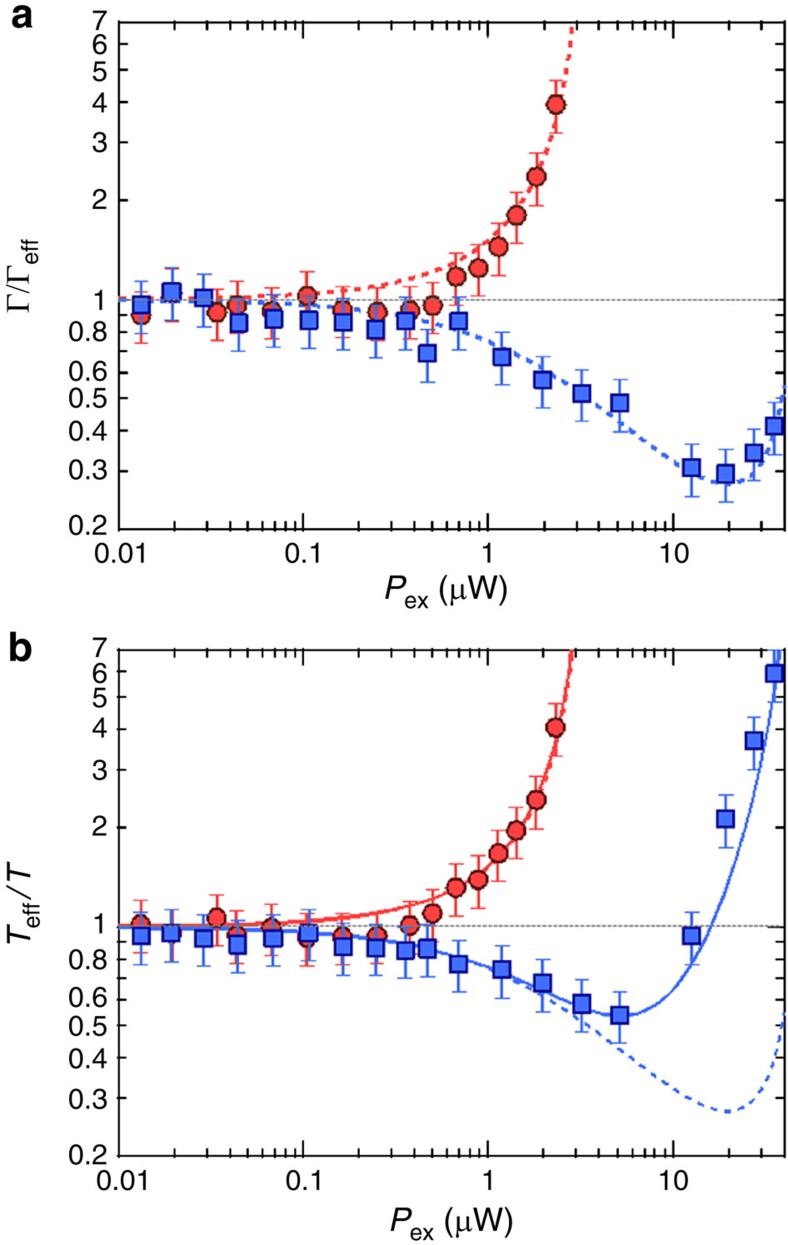
Comparison of the feedback gain and mode temperature. (**a**) *P*_ex_ dependence of feedback gain (Γ/Γ_eff_) for 1.5145 (red) and 1.5160, eV (blue). The broken line is a fit according to the modified equation (1), in which the opto-piezoelectric backaction (∇*F*_p_) with the linear *P*_ex_ dependence and the additional backaction force (−∇*F*_a_) with the 
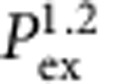
 dependence are both taken into account. (**b**) Laser power dependence of *T*_eff_/*T* for 1.5145 (red) and 1.5160, eV (blue). The solid line is a fit according to *T*_eff_/(*T*+*T*_b_)=Γ/Γ_eff_, where 
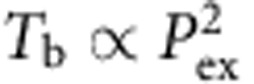
. The broken lines in **b** are identical to those in **a**. The error bars are based on the fitting of the Brownian noise power spectrum to the Lorentzian function.

Figure 4a shows the laser power dependence of the feedback gain, which corresponds to Γ/Γ_eff_, when the photon energy is set to 1.5145 (red detuning) and 1.5160, eV (blue detuning). For the red detuning, the gain increases with *P*_ex_ with the reduction of Γ_eff_, and for *P*_ex_>5 μW, the system enters the self-oscillation (negative damping) regime^9^. In contrast, for the blue detuning, increasing laser power decreases the gain for *P*_ex_<15 μW, following the theoretical form given by equation (1). In the stronger excitation regime (*P*_ex_≥15 μW), on the other hand, an additional effect appears, and it suppresses the damping caused by the opto-piezoelectric backaction in blue detuning. As a result, Γ/Γ_eff_ has a minimum at *P*_ex_=20 μW for 1.5160, eV (Fig. 4a). The overall data can be fitted by a modified equation (1), in which the additional backaction force with the opposite sign, −∇*F*_a_, is also taken into account (blue broken curve in Fig. 4a; Supplementary Note 2), though the origin of this additional effect is not clear at present.

The laser power dependence of *T*_eff_/*T* shows good agreement with that of Γ/Γ_eff_ in the excitation regime of *P*_ex_<3 μW (Fig. 4b) as described in equation (2). In contrast, for *P*_ex_≥3 μW, *T*_eff_/*T* deviates from Γ/Γ_eff_ (Fig. 4b). This indicates the existence of another effect that increases the noise temperature as *T*→*T*+*T*_b_. We found that *T*_b_ has *P*_ex_^2^ dependence, which appears when optical absorption occurs by band-gap excitation, being irrelevant to the exciton resonance (Supplementary Figs 3–5 and Supplementary Note 3). Modifying equation (2) as *T*→*T*+*T*_b_ results in a good agreement between *T*_eff_/*T* and Γ/Γ_eff_ (see the solid line in Fig. 4b). The non-negligible *T*_b_ draws back the minimum mode temperature, but a reduction by ∼0.5 is still achieved in this system.

## Discussion

As we have shown in equation (1), the gain of the opto-piezoelectric self-feedback depends on the mechanical quality factor, *Q*_m_. Therefore, the feedback efficiency will be improved in a higher *Q*_m_ resonator, which can be prepared, for example, by introducing tensile stress^28^. Hybridizing quantum dots^22^,^23^,^29^ or quantum wells^30^ that cause a sharper optical transition will also improve the coupling efficiency (Supplementary Note 6). Such improved systems open the way to novel optoelectromechanical integrated systems based on a semiconductor device platform. The on-chip hybridization with optical devices, such as like photo detectors and light emitting diodes,^31^ further expand the integration capability. This cavity-less coupling enables highly tunable vibration control of nanomechanical resonators via the addressable free-space optical access. This would, therefore, allow high-speed programmable manipulation of integrated nanomechanical systems, which expands their functionality in sensor, signal processing and information technologies^13^,^14^,^15^,^16^,^17^,^18^,^19^.

## Methods

### Fabrication

The cantilever (20 μm length and 14 μm width with 10-μm long 4-μm wide legs) was fabricated by photolithography and wet etching. It consists of 200-nm thick Si-doped Al_0.2_Ga_0.8_As (including a 5-nm thick GaAs cap layer) and 200-nm thick undoped GaAs, grown on a 3-μm thick Al_0.65_Ga_0.35_As sacrificial layer on a GaAs(001) substrate by the molecular beam epitaxy method. To avoid unintentional formation of an optical cavity between the cantilever and substrate, we removed the substrate below the free-standing part by selective cutting.

### Measurement

The cantilever was cooled with a cryocooler (ARS: DE-210). The Brownian motion of the cantilever was measured by He:Ne laser interferometry (NeoArk:MLD-221) with a spectrum analyser (HP:89410A). A wavelength-tunable cw Ti:Sa laser (Spectra Physics:3900S) was used for the optical excitation. The He:Ne laser was focused near the free-edge to detect the mechanical motion sensitively, while the Ti:Sa laser was focused on the leg where larger strain is obtained.

## Additional information

**How to cite this article:** Okamoto, H. *et al*. Cavity-less on-chip optomechanics using excitonic transitions in semiconductor heterostructures. *Nat. Commun.* 6:8478 doi: 10.1038/ncomms9478 (2015).

## Supplementary Material

Supplementary Information Supplementary Figures 1-5, Supplementary Notes 1-6 and Supplementary References
