# Supplementary material for: Cavity-less on-chip optomechanics using excitonic transitions in semiconductor heterostructures
Source: Nat Commun. 2015 Oct 19;6:8478. doi: 10.1038/ncomms9478 (PMC4634130; doi:10.1038/ncomms9478)
Supplement: Supplementary Information — Supplementary Figures 1-5, Supplementary Notes 1-6 and Supplementary References [file ncomms9478-s1.pdf]

Supplementary Figures

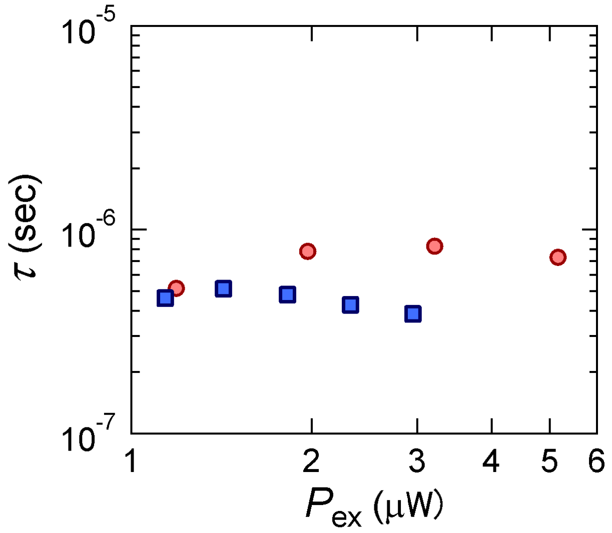

Supplementary Figure 1: Extracted delay time τ of the opto-piezoelectric backaction with respect to laser power for 1.5145 (red circles) and 1.5160 eV (blue squares).

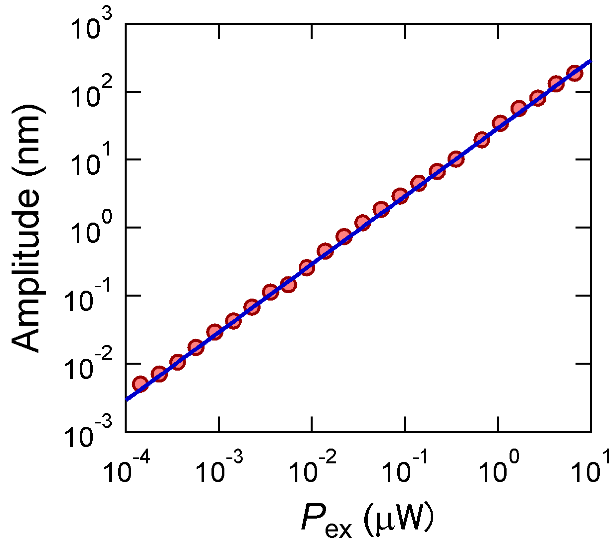

Supplementary Figure 2: Laser power dependence of the vibration amplitude at the resonance frequency measured for 1.5152 eV (at the exciton peak) under the modulated illumination.

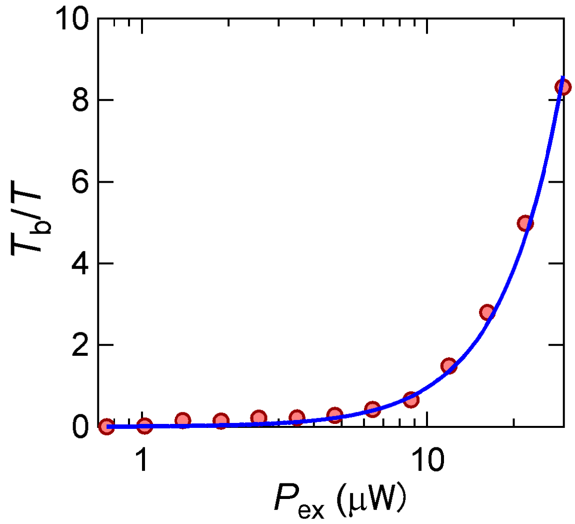

Supplementary Figure 3: Laser power dependence of T_b/T measured for 1.521 eV (above the band-gap energy) under the constant illumination. The solid curve shows the fitting with $T_b/T = B_T P_{ex}^2$, where the resultant coefficient is $B_T = 0.01$ μW^{-2}.

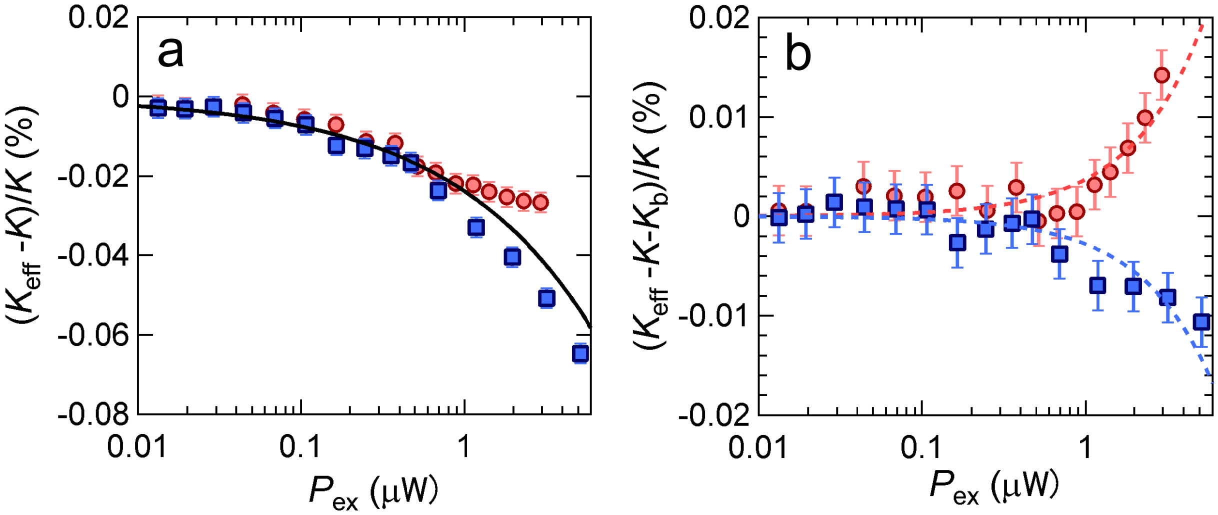

Supplementary Figure 4: Laser power dependence of the spring constant. a, Shift of the spring constant, $(K_{\rm eff} - K)/K$, with respect to laser power for 1.5145 (red circles) and 1.5160 eV (blue squares). The solid curve shows the offset shift, K_b/K, with the $P_{ex}^{0.5}$ dependence. b, Shift of the spring constant by the opto-piezoelectric backaction, $(K_{\rm eff} - K - K_b)/K$, with respect to laser power for 1.5145 (red circles) and 1.5160 eV (blue squares), from which offset shift K_b/K is subtracted. The error bars are based on the fitting of the Brownian noise power spectrum to the Lorentzian function.

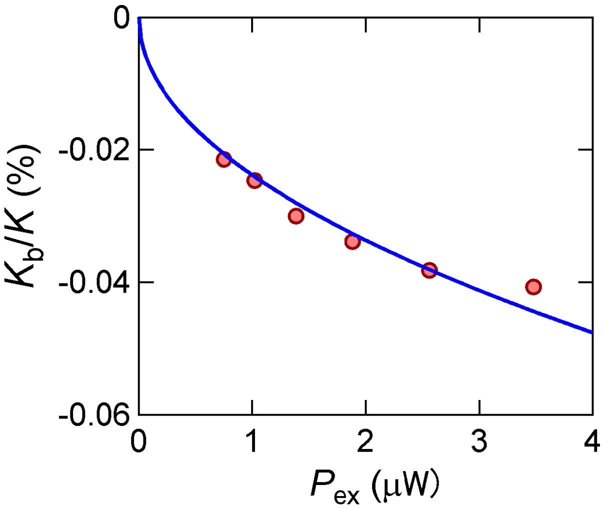

Supplementary Figure 5: Laser power dependence of the shift in the spring constant, K_b/K, measured for 1.521 eV (above the band-gap energy). The solid curve follows $K_\mathrm{b}/K = -B_\mathrm{K} P_\mathrm{ex}^{0.5}$, where $B_\mathrm{K} = 2.4 \times 10^{-4}$ μW$^{-0.5}$. It is identical to the solid curve in Supplementary Figure 4a.

Supplementary Note 1: Opto-piezoelectric backaction

We begin by introducing an equation of motion for the cantilever, in which the opto-piezoelectric backaction via e-h pairs is taken into account under constant illumination. The derivation is similar to the case of bolometric backaction[1] and described in detail in Supplementary Ref. 2. Assuming that backaction force F_p acts in time delay τ with respect to the motion of the cantilever, the equation of motion can be given by

$$m\ddot{z}(t) + m\Gamma\dot{z}(t) + Kz(t) = F_\mathrm{th}(t) + F_\mathrm{p}(z_0) + \int_{-\infty}^{t} \frac{dF_\mathrm{p}(z(t'))}{dt'} h(t-t')dt', \quad (1)$$

where z is the displacement of the cantilever, z_0 is the averaged position, m is the effective mass, Γ $(= \omega_0/Q_\mathrm{m})$ is the bare damping factor, K $(= m\omega_0^2)$ is the bare spring constant, F_th is the thermal Langevin force, $F_\mathrm{p}(z(t))$ is the opto-piezoelectric backaction force, and $h(t)$ is the response function given by $h(t) = 1 - \exp(-t/\tau)$. Here, $F_\mathrm{p}(z_0)$ just leads to a static shift of the cantilever's average position. Thus, by selecting the new average position for z, it can be dropped in the following derivation.[1,2] The z-dependent component, $F_\mathrm{p}(z(t))$, is caused by the strain effect via the deformation potential and it modifies Γ and K. Under the small displacement approximation, Supplementary Equation 1 can be transformed in the frequency domain as[1,2]

$$-m\omega^2 z_\omega + i\omega m\Gamma_\mathrm{eff} z_\omega + K_\mathrm{eff} z_\omega = F_{\mathrm{th},\omega}, \quad (2)$$

where

$$\Gamma_{\text{eff}} = \Gamma\left(1+\frac{Q_m\omega_0\tau}{1+\omega^2\tau^2}\frac{\nabla F_{\text{p}}}{K}\right) \tag{3a}$$

$$K_{\text{eff}} = K\left(1-\frac{1}{1+\omega^2\tau^2}\frac{\nabla F_{\text{p}}}{K}\right) \tag{3b}$$

and ∇F_{p} $(= \partial F_{\text{p}}(z)/\partial z|_{z=z_0})$ is the force gradient around the averaged position. The frequency component of the amplitude is given with the modified frequency ω_{eff} and Γ_{eff} by[1,2]

$$z_\omega = \frac{F_{\text{th},\omega}}{m(\omega_{\text{eff}}^2-\omega^2+i\omega\Gamma_{\text{eff}})}. \tag{4}$$

Here, it can be assumed that $F_{\text{p}}(z)$ is proportional to the number of electron-hole (e-h) pairs $N(z)$ that contribute to the opto-piezoelectric backaction. Thus

$$F_{\text{p}}(z) = C_{\text{piezo}}N(z), \tag{5}$$

where C_{piezo} is the piezoelectric coefficient. $N(z)$ depends on the excitation power P_{ex} and the energy detuning E from the exciton resonance energy E_{e}. Thus, Supplementary Equation 5 is more generally written as

$$F_{\text{p}}(E,P_{\text{ex}},z) = C_{\text{piezo}}N(E,P_{\text{ex}},z). \tag{6}$$

For the fixed photon energy,

$$\nabla F_{\text{p}}(P_{\text{ex}},z) = \frac{\partial F_{\text{p}}(P_{\text{ex}},z)}{\partial z}|_{z=z_0} = C_{\text{piezo}}\frac{\partial N(P_{\text{ex}},z)}{\partial z}|_{z=z_0}. \tag{7}$$

For the fixed laser power,

$$\nabla F_{\text{p}}(E,z) = \frac{\partial F_{\text{p}}(E,z)}{\partial z}|_{z=z_0} = C_{\text{piezo}}\frac{\partial N(E,z)}{\partial E}\frac{\partial E_{\text{e}}}{\partial z}|_{z=z_0} = C_{\text{piezo}}C_{\text{deform}}\frac{\partial N(E,z)}{\partial E}, \tag{8}$$

where $C_{\text{deform}} = \frac{\partial E_{\text{e}}}{\partial z}|_{z=z_0}$, which is related to the deformation potential (-8.8 eV in GaAs)[3] and estimated to be $C_{\text{deform}} \sim 9$ μeV/nm as described in Supplementary Note 5. Assuming $N(E)$ is proportional to the optical absorption, *i.e.* to the intensity I of the photoluminescence excitation (PLE) spectrum, then $\nabla F_{\text{p}}(E,z) \propto dI/dE$. That is, the backaction is proportional to the slope of the PLE spectrum. Thus, Supplementary Equation 3a has the form

$$\Gamma_{\text{eff}} = \Gamma\left(1+C\frac{dI}{dE}\right), \tag{9}$$

where coefficient C has a negative sign for red-detuning and a positive sign for blue-detuning.

For the modulated illumination, on the other hand, opto-piezoelectric force $F_{\text{p}}(z(t),t)$ is given as

$$F_{\text{p}}(z(t),t) = [1+\varepsilon(t)]F_{\text{p}}(z(t)), \tag{10}$$

where $\varepsilon(t)$ is the modulation amplitude. The equation of motion is then given by[1,2]

$$m\ddot{z}(t) + m\Gamma\dot{z}(t) + Kz(t) = F_{\mathrm{th}}(t) + \int_{-\infty}^{t} \left(\frac{\partial F_{\mathrm{p}}}{\partial t'} + \frac{\partial F_{\mathrm{p}}}{\partial z}\frac{\partial z}{\partial t'} \right) h(t-t')dt', \qquad (11)$$

where F_{th} is negligibly small compared with the driving force F_{p}, and therefore it can be ignored. The frequency component of the amplitude is approximated by[1,2]

$$z_{\omega} = \frac{\varepsilon_{\omega} F_{\mathrm{p},\omega}}{m(1+i\omega\tau)(\omega_{\mathrm{eff}}^2 - \omega^2 + i\omega\Gamma_{\mathrm{eff}})}, \qquad (12)$$

which shows the resonance amplitude is proportional to F_{p}.

The experimental data under the modulated illumination reveals that the resonance amplitude linearly increases with increasing laser power P_{ex} in the excitation regime of $P_{\mathrm{ex}} < 10$ μW (Supplementary Figure 2). This indicates $F_{\mathrm{p}} \propto P_{\mathrm{ex}}$, suggesting the band flattening caused by the screening effect is negligibly small in this power regime. This also suggests $\nabla F_{\mathrm{p}} \propto P_{\mathrm{ex}}$, so that Supplementary Equations 3a and 3b have the forms

$$\Gamma_{\mathrm{eff}} = \Gamma\left(1 + C_{\Gamma} P_{\mathrm{ex}}\right) \qquad (13a)$$

$$K_{\mathrm{eff}} = K\left(1 - C_{\mathrm{K}} P_{\mathrm{ex}}\right), \qquad (13b)$$

where C_{Γ} and C_{K} are coefficients with a negative sign for red-detuning and a positive sign for blue-detuning.

From the equipartition theorem, the damping factor is related to the mode temperature as[1,2]

$$\frac{T_{\mathrm{eff}}}{T} = \frac{\Gamma}{\Gamma_{\mathrm{eff}}}, \qquad (14)$$

where T_{eff} can be experimentally extracted from the area of Brownian displacement noise power spectrum. Therefore, the theoretical form of the normalized mode temperature is given by $T_{\mathrm{eff}}/T = \Gamma/\Gamma_{\mathrm{eff}} = (1 + CdI/dE)^{-1}$. This means the mode temperature reflects the slope of the PLE spectrum. We experimentally confirm this tendency in Fig. 3c in the main text.

Supplementary Note 2: Additional feedback in the strong excitation regime

The laser power dependence of the damping factor (Fig. 4a in the main text) reveals that the additional backaction with the opposite sign, $-\nabla F_{\mathrm{a}}$, appears in the strong excitation regime of $P_{\mathrm{ex}} \geq 15$ μW. This suppresses the opto-piezoelectrically induced damping in the blue-detuning condition. The overall experimental data below $P_{\mathrm{ex}} < 40$ μW can be fitted with the form

$$\Gamma_{\mathrm{eff}} = \Gamma\left(1 + C_{\Gamma} P_{\mathrm{ex}} - A_{\Gamma} P_{\mathrm{ex}}^{r}\right), \qquad (15)$$

where A_{Γ} is the coefficient for the additional backaction term and r is the exponent. The fitting provides an non-integer value for r $(= 1.2)$, so it does not straightforwardly help

us to understand the phenomenon in the high-power regime. The band screening effect is also included in the high-power regime. Such nonlinear optical properties prevent simple understanding of the additional backaction effect. Therefore, the origin of the additional backaction is not clear at present. The bolometric backaction might be the source,[1,4] which comes from the local thermal expansion that would dominantly occur in the GaAs layer by optical absorption. The lattice expansion in the GaAs layer tends to bend the cantilever in the opposite direction with respect to the opto-piezoelectric force. It could therefore be the source of $-\nabla F_{\mathrm{a}}$.

Supplementary Note 3: The effect of band-gap absorption

With laser irradiation, there exists a noise source that directly increases the mode temperature. This effect can be described by adding the noise force $F_{\mathrm{b}}(t)$ to Supplementary Equation 1. Supplementary Equation 14 is modified by $T \rightarrow T + T_{\mathrm{b}}$ as

$$\frac{T_{\mathrm{eff}}}{T} = \frac{\Gamma}{\Gamma_{\mathrm{eff}}}\left(1 + \frac{T_{\mathrm{b}}}{T}\right), \tag{16}$$

where T_{b} is the noise temperature coming from $F_{\mathrm{b}}(t)$. The experimental result (Fig. 4b in the main text) shows T_{b} is negligible for $P_{\mathrm{ex}} < 3$ μW but not for the stronger excitation. T_{b} is not related to the exciton resonance but comes from the band-gap absorption in GaAs, which is confirmed by setting the photon energy above the band-gap energy. The laser-power dependence of the area of the displacement noise power spectrum for $P_{\mathrm{ex}} = 1.521$ eV exhibits the relation $T_{\mathrm{b}}/T = B_{\mathrm{T}} P_{\mathrm{ex}}^2$, where $B_{\mathrm{T}} = 0.01$ $\mu\mathrm{W}^{-2}$, as shown in Supplementary Figure 3. Supplementary Equation 16 thus has the form

$$\frac{T_{\mathrm{eff}}}{T} = \frac{\Gamma}{\Gamma_{\mathrm{eff}}}\left(1 + B_{\mathrm{T}} P_{\mathrm{ex}}^2\right). \tag{17}$$

The experimental data of T_{eff}/T can be well traced by Supplementary Equation 17 as shown in Fig. 4b in the main text. Note that B_{T} becomes an order of magnitude smaller when the photon energy is set below the band-gap energy (*e.g.* $B_{\mathrm{T}} = 8 \times 10^{-4}$ $\mu\mathrm{W}^{-2}$ for $P_{\mathrm{ex}} = 1.512$ eV), indicating T_{b} originates from band-gap absorption.

The effect of band-gap absorption also appears through the offset shift in the spring constant. Supplementary Figure 4a shows the experimentally measured change in the spring constant, $(K_{\mathrm{eff}} - K)/K$, as a function of P_{ex} for red-detuning (1.5145 eV) and blue-detuning (1.5160 eV) from the exciton resonance. It shows the negative offset shift (black curve in Supplementary Figure 4a) plus the positive shift for the red-detuning (red plots) or the negative shift for the blue-detuning (blue plots). By subtracting the offset shift, we can extract the change in the spring constant due to the opto-piezoelectric backaction as shown in Supplementary Figure 4b. The optically modified spring constant, K_{eff}, therefore contains two contributions: the offset shift caused by the band-gap absorption, K_{b}/K, and the opto-piezoelectric backaction given by Supplementary Equation 3b, *i.e.*,

$$K_{\mathrm{eff}} = K\left(1 + \frac{K_{\mathrm{b}}}{K} - \frac{1}{1+\omega^2\tau^2}\frac{\nabla F_{\mathrm{p}}}{K}\right). \tag{18}$$

The experimental result shows that the offset shift is $P_{ex}^{0.5}$ dependent, while the shift by the opto-piezoelectric backaction is proportional to P_{ex}. Thus, Supplementary Equation 18 has the form

$$K_{eff} = K\left(1 - B_K P_{ex}^{0.5} - C_K P_{ex}\right), \tag{19}$$

where coefficient C_K is negative for red-detuning and positive for blue-detuning. The data fitting provides the coefficients as $B_K = 2.4 \times 10^{-4}\ \mu W^{-0.5}$ and $C_K = \pm 3 \times 10^{-5}\ \mu W^{-1}$. Note that the offset shift in Supplementary Figure 4a shows good agreement with the laser power dependence of the spring constant measured for $P_{ex} = 1.521$ eV (above the band-gap energy, see Supplementary Figure 5). This also verifies that K_b originates from band-gap absorption.

High-power excitation potentially causes ohmic loss and thermoelastic damping in addition to the shift of the spring constant, but neither of them leads to the reduction of the damping factor as observed in Fig. 4a. Therefore, it is considered that the reduction of the damping factor due to the additional backaction is not linked to the above mentioned frequency shift caused by the band-gap absorption.

Supplementary Note 4: Delay time of the opto-piezoelectric backaction

Delay time τ of the opto-piezoelectric backaction can be extracted from the change in the damping factor and spring constant as[2]

$$\tau = \frac{\Gamma - \Gamma_{eff}}{\omega_{eff}^2 - \omega^2}. \tag{20}$$

Supplementary Figure 1 shows the laser power dependence of τ for red-detuning (1.5145 eV) and blue-detuning (1.5160 eV) from the exciton energy, which is extracted from Supplementary Figure 4b and Fig. 4a in the main text. The result shows τ is in the range of 0.4 - 0.8 μs, which is close to $\omega_0^{-1} = 2\pi f_0^{-1} = 0.41\ \mu$s. This means the backaction force is almost $\pi/2$-phase shifted with respect to the oscillation phase, which results in the effective self-feedback effect.[2] Note also that the measured τ is on the order of typical non-radiative recombination lifetime in GaAs.[2] This also suggests that the time delay comes from the spatial separation of electrons and holes in the cantilever.

Supplementary Note 5: Optomechanical coupling strength

In our system, optomechanical coupling is based on the strain-induced modulation of the exciton energy, E_e. As in Supplementary Refs. 5 and 6, we can extract the opto-mechanical coupling parameter, $\partial E_e/\partial z$, which represents the exciton energy shift per displacement and corresponds to the coefficient C_{deform} in Supplementary Equation 8. In our cantilever, the free-edge displacement of 0.1 nm leads to strain of 1×10^{-7} at the leg. Taking into account this value and the deformation potential of GaAs ($\sim$9 eV), $\partial E_e/\partial z$ is estimated to be $\sim$9 μeV/nm. This is similar to the value in a quantum dot-embedded nanowire

system in Supplementary Ref. 6 ($\partial E_e/\partial z = 10\ \mu$eV/nm), getting a benefit from the large deformation potential effect in GaAs. From the motional mass, $m = 1.6 \times 10^{-13}$ kg, and the mechanical resonance frequency, $\omega_0 = 2\pi\times$ 386.68 kHz, the zero-point motion, $z_{\rm zpf}$, is estimated to be 1.2×10^{-14} m. Thus, we obtain the opto-mechanical coupling strength as $g_0 = 2\lambda = (1/\hbar)\partial E_e/\partial z \cdot z_{\rm zpf} = 2\pi\times$ 25 kHz. The ratio of this parameter to the mode frequency, $g_0/\omega_0 = 0.07$, is smaller than the unity, *i.e.*, smaller than the ultrastrong coupling limit,[5] but still comparable with the value in Supplementary Ref. 6 ($g_0/\omega_0 = 0.17$).

Supplementary Note 6: Theoretical limit of opto-piezoelectric cooling

The theoretical limit of opto-piezoelecric cooling is given by the same form as the bolometric cooling.[1] Cooling stops when the static spring constant, $K_{\rm eff}(\omega = 0)$, becomes zero, *i.e.*, $K_{\rm eff} = K(1 - \nabla F_p/K) = 0$. Considering $\omega_0\tau \sim 1$, the theoretical limit of the effective temperature is given by $T_{\rm eff,lim}/T = 1/(1 + Q_m/2)$. Here, the mechanical quality factor of our cantilever is Q_m = 5,600. This indicates the cooling limit by the opto-piezoelectric backaction is further beyond the observed minimum temperature, $T_{\rm eff}/T \sim 0.5$. As described in Supplementary Note 2, the additional feedback in the high-power regime prevents further cooling toward the theoretical limit, where the origin of the additional effect is not clear at present. But even with this additional effect we can expect that the higher Q_m leads to the lower $T_{\rm eff}$ because $T_{\rm eff,lim} \propto Q_m^{-1}$. In the GaAs-based mechanical system, two orders of magnitude higher Q_m can be prepared by introducing tensile stress.[7,8] Therefore, two orders of magnitude lower $T_{\rm eff}$ is achievable. This scheme is not limited to the top-down-fabricated cantilevers but can also be adapted to bottom-up-fabricated III-V semiconductor nanowires as in Supplementary Refs. 5 and 6 if the built-in potential is successfully installed. An advantage in such systems is the sharp exciton resonance (Q_e = 21,400 in Ref. 5 and Q_e = 13,300 in Ref. 6), which Q_e is an order of magnitude larger than that of the present cantilever (Q_e = 1,500). The nanowire in Supplementary Ref. 5 also results in an order of magnitude larger optomechanical coupling parameter, $\partial E_e/\partial z = 166\ \mu$eV/nm. The larger coupling parameter as well as the larger Q_e will lead to orders of magnitude larger backaction effect.

Supplementary References

1. Metzger, C., Favero, I., Ortlieb, A. & Karrai, K. Optical self cooling of a deformable Fabry-Perot cavity in the classical limit. *Phys. Rev. B* **78**, 035309 (2008).
2. Okamoto, H. *et al.* Carrier-mediated optomechanical coupling in GaAs cantilevers. *Phys. Rev. B* **84**, 014305 (2011).
3. Cardona, M. & Christensen, N. E. Acoustic deformation potentials and heterostructure band offsets in semiconductors. *Phys. Rev. B.* **35**, 6182-6194 (1987).
4. Usami, K. *et al.* Optical cavity cooling of mechanical modes of a semiconductor nanomembrane. *Nature Phys.* **8**, 168-172 (2012).

[5] Yeo, I. *et al.* Strain-mediated coupling in a quantum dot-mechanical oscillator hybrid system. *Nature Nanotech.* **9**, 106-110 (2014).

[6] Montinaro, M. *et al.* Quantum dot opto-mechanics in a fully self-assembled nanowire. *Nano Lett.* **14**, 4454-4460 (2014).

[7] Yamaguchi, H. *et al.* Improved resonance characteristics of GaAs beam resonators by epitaxially induced strain. *Appl. Phys. Lett.* **92**, 251913 (2008).

[8] Onomitsu, K., Mitsuhara, M., Yamamoto, H., & Yamaguchi, H. Ultrahigh-Q micromechanical resonators by using epitaxially induced tensile strain in GaNAs. *Appl. Phys. Exp.* **6**, 111201 (2013).
